# Distinct host-response signatures in circulatory shock: a narrative review

**DOI:** 10.1186/s40635-023-00531-5

**Published:** 2023-08-18

**Authors:** Sabri Soussi, Claudia dos Santos, Jacob C. Jentzer, Alexandre Mebazaa, Etienne Gayat, Janine Pöss, Hannah Schaubroeck, Filio Billia, John C. Marshall, Patrick R. Lawler

**Affiliations:** 1 Department of Anesthesia and Pain Management, University Health Network (UHN), Women’s College Hospital, University of Toronto, Toronto Western Hospital, 399 Bathurst St, ON M5T 2S8 Toronto, Canada; 2 St Michael’s Hospital, Keenan Research Centre for Biomedical Science and Institute of Medical Sciences, University of Toronto, Toronto, ON Canada; 3 Department of Cardiovascular Medicine, Mayo Clinic Rochester, Rochester, MN 55905 USA; 4 Department of Anesthesiology, Critical Care, Lariboisière-Saint-Louis Hospitals, DMU Parabol, AP–HP Nord; Inserm UMR-S 942, Cardiovascular Markers in Stress Conditions (MASCOT), University of Paris, Paris, France; 5 Department of Internal Medicine/Cardiology, Heart Center Leipzig at the University of Leipzig, Strümpellstraße, 39 04289 Leipzig, Germany; 6 Department of Intensive Care Medicine, Department of Internal Medicine and Pediatrics, Ghent University Hospital, Ghent University, Corneel Heymanslaan 10, 9000 Ghent, Belgium; 7 Peter Munk Cardiac Centre, University Health Network, University of Toronto, Toronto, ON Canada; 8 McGill University Health Centre, McGill University, Montreal, QC Canada; 9 Ted Roger’s Center for Heart Research, University Health Network, University of Toronto, Toronto, ON Canada

## Abstract

Circulatory shock is defined syndromically as hypotension associated with tissue hypoperfusion and often subcategorized according to hemodynamic profile (e.g., distributive, cardiogenic, hypovolemic) and etiology (e.g., infection, myocardial infarction, trauma, among others). These shock subgroups are generally considered homogeneous entities in research and clinical practice. This current definition fails to consider the complex pathophysiology of shock and the influence of patient heterogeneity. Recent translational evidence highlights previously under-appreciated heterogeneity regarding the underlying pathways with distinct host-response patterns in circulatory shock syndromes. This heterogeneity may confound the interpretation of trial results as a given treatment may preferentially impact distinct subgroups. Re-analyzing results of major ‘neutral’ treatment trials from the perspective of biological mechanisms (i.e., host-response signatures) may reveal treatment effects in subgroups of patients that share treatable traits (i.e., specific biological signatures that portend a predictable response to a given treatment). In this review, we discuss the emerging literature suggesting the existence of distinct biomarker-based host-response patterns of circulatory shock syndrome independent of etiology or hemodynamic profile. We further review responses to newly prescribed treatments in the intensive care unit designed to personalize treatments (biomarker-driven or endotype-driven patient selection in support of future clinical trials).

## Introduction

Circulatory shock incurs a high mortality, with minimal incremental improvements in survival demonstrated by clinical trials in the last two decades [1–4]. For the purposes of research and clinical practice, circulatory shock is defined as hypotension associated with tissue hypoperfusion, and traditionally subclassified based on etiology (e.g., trauma, infection, myocardial infarction among others) and hemodynamic profile (e.g., vasodilatory, cardiogenic, hypovolemic). Each subtype is generally treated as a homogenous clinical syndrome [5, 6].

The initial step in the management of circulatory shock is generally to control the cause of shock (e.g., anti-microbials for sepsis, surgical or interventional treatment for bleeding, percutaneous coronary intervention for myocardial infarction). Medical therapy is provided in the form of volume replacement, transfusions, and vasopressors/inotropes to correct hypovolemia, anemia, hypotension and low cardiac output. In the case of severe organ dysfunction, patients receive supportive therapies, including renal replacement therapy, mechanical ventilation, and/or mechanical circulatory support (e.g., veno-arterial extracorporeal membrane oxygenation) [5]. The ‘missing link’ in the current treatment strategy/algorithm is personalized/targeted intervention to address the underlying host response driving circulatory shock and organ dysfunction [7].

Emerging translational evidence demonstrates the existence of variability in the host response to circulatory shock syndromes and the existence of potential common ‘treatable traits’ (i.e., specific biological signatures that portend a predictable response to a given treatment) within subgroups of patients across different forms of shock. Conserved biological signatures occurring across diverse shock subtypes include combinations of endothelial dysfunction, inflammatory cell activation, reactive oxygen species formation, immune dysregulation, neurohormonal dysregulation, activation of coagulation, and fibrinolysis [8–11].

It has been hypothesized that heterogeneity in a given etiology or hemodynamic profile of circulatory shock may dilute a demonstrable benefit of specific interventions when applied broadly in clinical trials due to diverse individual treatment response profiles [4, 12]. It has also been hypothesized that there are shared host response and organ injury patterns across different circulatory shock etiologies (i.e., insults) that may have comparable treatment responsiveness [8, 9, 13, 14].

In this narrative review, we overview the current literature related to **(i)** distinct and common biological patterns of host response in circulatory shock syndromes and **(ii)** their response to different established (e.g., corticosteroids, vasopressors/inotropes) or novel interventions (e.g., immune modulation, antibody treatment). This paper is a narrative review from the 2022 Critical Care Clinical Trialists (3CT) Workshop expert panel for circulatory shock endotyping (https://www.3ctmeeting.com/). Our aim is to highlight the main studies in the field and not to provide an exhaustive review of the literature.

## The heterogeneity of the host response within clinical circulatory shock syndromes

Biomolecular heterogeneity is not reflected by non-specific clinical syndromic criteria for patients with critical illness, specifically those with shock. Even if standardized diagnostic criteria for sepsis/septic shock, cardiogenic shock and hemorrhagic shock have evolved over the years, yet substantial underlying heterogeneity exists within populations of patients meeting these definitions.

Here, we summarize the main studies using readily available clinical and biological data and/or omics-based biomarkers (e.g., proteomics, metabolomics, transcriptomics) to unravel host response heterogeneity within distinct circulatory shock syndromes.

### Sepsis and septic shock

In a retrospective analysis of sepsis/septic shock cohorts and clinical trials, Seymour et al*.* identified four distinct clinical phenotypes of sepsis using readily available clinical and biological data at hospital admission [15]. The phenotypes were identified using latent class analysis and did not equate with traditional patient groupings (e.g., organ dysfunction, severity of illness, site of infection). The clinical phenotypes correlated with host-response patterns, clinical outcomes, and response to tested interventions.

In a prospective observational cohort of 288 sepsis/septic shock adult patients in Uganda, Cummings et al. used unsupervised clustering of 14 soluble host immune mediators, reflective of key domains of sepsis immunopathology (innate and adaptive immune activation, endothelial dysfunction, fibrinolysis), and to whole-blood RNA-sequencing data to identify immune and transcriptional subtypes [16]. The authors identified distinct immune subtypes with a different activation of proinflammatory innate and adaptive immune pathways, with T cell exhaustion, aberrant NK cell expansion and oxidative stress in the hyperinflammatory subtype. Host response subtypes defined by upregulation of the aforesaid pathways were associated with disseminated HIV-associated tuberculosis, more severe organ dysfunction and worse outcomes. These results highlight the presence of host- and pathogen-driven biological features of septic patients.

In the study of Davenport et al. only ~ 60% of patients with pneumosepsis had “classic” immune activation (as many as 40% manifested an immunosuppressed response). In this study, the authors assayed peripheral blood leukocyte gene expression in septic shock patients. The authors used a clustering approach to identify two sepsis response signatures (SRS). SRS-1 had an immunosuppressed phenotype and worse outcome [17].

### Cardiogenic shock

Zweck et al. recently used *k*-means clustering (a non-model-based clustering technique) to identify three subclasses/phenotypes in a multicenter cardiogenic shock cohort [18]. The three reported phenotypes were determined based on six admission biological variables (white blood cell count, platelet count, estimated glomerular filtration rate, alanine aminotransferase, lactate, bicarbonate) and were labeled “noncongested”, “cardiorenal”, and “hemometabolic”. Interestingly, these biological phenotypes were associated with mortality (higher risk of mortality in the hemometabolic phenotype) independently from the Society for Cardiovascular Angiography and Interventions (SCAI) staging of shock severity.

These three clusters were replicated by Jentzer et al. with the same six admission laboratory variables in 1498 cardiogenic shock patients, finding differences in echocardiographic markers of cardiac function and long-term survival between groups [19].

In an exploratory study of 107 patients with ST-segment-elevation myocardial infarction and acute heart failure (i.e., cardiogenic pre-shock), peripheral leukocyte gene expression patterns (mRNA expression data) were used to identify host-response endotypes. Hierarchical clustering grouped patients in two endotypes based on pathway variability in mediators of inflammation, immune function. Demographic and clinical characteristics did not significantly different across host-response endotypes, suggesting that molecular profiling might be incremental to clinical classifiers alone [20].

Last and not least, Scolari et al. reported that cardiogenic shock patients have a higher frequency of clonal haematopoiesis (i.e., specific gene mutations, notably in TET2 and ASXL1, in haematopoietic stem cells which lead to clonal expansion) than in patients with ambulatory heart failure matched for age, sex, ejection fraction, and heart failure etiology [21]. This condition was also associated with reduced survival and dysregulation of circulating inflammatory cytokines in cardiogenic shock patients with clonal haematopoiesis.

### Major trauma and hemorrhagic shock

In an analysis of a prospective cohort study including 102 severe trauma patients with hemorrhagic shock, Brakenridge et al*.* used a clustering approach to identify distinct immunologic endotypes [22]. Multiple biomarkers were used to assess the magnitude of hyper-inflammation and immunosuppression over time [e.g., IL-6, IL-8, IL-10, granulocyte colony-stimulating factor (G-CSF), granulocyte–macrophage colony-stimulating factor (GM-CSF), monocyte chemoattractant protein-1 (MCP-1), interferon-γ-inducible protein 10 (IP-10 [CXCL10]), IL-17 alpha and soluble programmed death ligand 1 (sPD-L1)]. They identified three distinct immunologic endotypes (iA, iB, and iC), with a different association with clinical trajectory. The endotype iB with persistent inflammation and immunosuppression (40% of the studied population) was strongly associated with persistent organ dysfunction, increased infections, and prolonged ICU length of stay.

Cyr et al*.* used untargeted metabolomics and plasma immune circulating markers in 86 severely ill trauma patients to identify distinct host-response subclasses [23]. Three host-response subclasses were identified: nonresponders (no time-dependent change in sphingolipids), sphingosine/sphinganine-enhanced, and glycosphingolipid-enhanced. The nonresponder subclass was characterized by more organ dysfunction, longer mean length of stay and higher circulating levels of proinflammatory immune mediators despite similar severity of trauma as evaluated by Injury Severity Scores. The findings may suggest that immunometabolic response signatures may be present among patients with severe trauma.

### Shared host-response patterns across different circulatory shock etiologies

More and more evidence suggests that the host response to many forms of injury is shared across distinct circulatory shock syndromes. The same biological signatures (e.g., endothelial dysfunction, inflammation, immune response) were highlighted in circulatory shock with different etiologies using a single-biomarker or a multiple-biomarkers approach.

### Single-biomarker approach

It has been reported in both sepsis/septic shock and cardiogenic shock that levels and rapid changes in circulating bio-adrenomedullin (a marker of endothelial dysfunction involved in vasodilatation and induction of angiogenesis) on admission are associated with worse hemodynamics and organ dysfunction independently from severity of chronic and acute illness and initial lactate level [24, 25].

Similarly, high circulating levels of angiopoietin-2 (a regulator of endothelial cell function) were reported as associated with organ dysfunction and mortality in distinct populations with cardiogenic shock, septic shock, and traumatic hemorrhagic shock independently from age, comorbidities, and severity scores on admission [26–28].

These results support the suggestion of Johansson et al. of a mechanistic link between sympatho-adrenal hyperactivation in circulatory shock independently from the insult and the endothelial phenotype. They defined a potential unifying pathophysiologic mechanism linked to poor outcome as shock-induced endotheliopathy [8].

### Multiple-biomarkers approach

In the Inflammation and the Host Response to Injury large-scale collaborative research program (Glue Grant), the investigators described the circulating leukocyte transcriptome in critically injured patients with circulatory shock [29]. They compared genome-wide expression from adult patients with trauma (*n* = 167) with matched (age, sex and ethnicity) healthy subjects and with 133 severely burned patients or 4 healthy adult subjects administered low-dose bacterial endotoxin. Despite different insults, the early genomic changes were highly comparable (> 80% of the cellular functions and pathways) between blunt trauma patients, burns, or stressors. These findings demonstrate a common host-response pattern reflective of the large overlap in upstream receptors and signaling intermediates activated by each condition (e.g., release of damage-associated molecular patterns (DAMPs), Toll-like receptor 4 (TLR4), alarmins).

Venet et al*.* recently described common injury-induced immune profiles in a large cohort of critically ill patients with different etiologies (e.g., sepsis, severe trauma, major surgery) [30]. The authors used an immunomonitoring panel (i.e., a combined monitoring of 30 circulating markers of pro/antiinflammatory, innate, and adaptive immune responses incorporating data from flow cytometry, functional assays, and protein- and messenger RNA-level measurements) to detect a delayed (seven days after admission) injury-acquired immunodeficiency in a subclass of severely injured patients independently from the admission diagnosis. This subclass of patients with profound immunosuppression was associated with a greater risk of secondary infections independently from exposure to invasive procedures.

Braga et al*.* reported in the ShockOmics cohort (*n* = 37) a similar pattern of differential expression of genes coding for inflammatory and immunoglobulin proteins among patients with sepsis and cardiogenic shock. The overlap in biological patterns suggests shared mechanistic signatures between the two critical illness syndromes [14].

Chen et al*.* used an unsupervised clustering approach across three etiologies of critically ill patients (i.e., severe trauma, sepsis, burn injury) to identify distinct molecular subclasses based on single cell transcriptomic patterns in circulating leukocytes. The authors identified three clusters reflecting dysregulation in genes involved in DNA repair and RNA processing between the etiologies (i.e., shared host-response patterns) with different associated clinical outcomes [31].

## Predictive enrichment to overcome circulatory shock heterogeneity

The U.S. Food and drug administration (FDA) defines predictive enrichment in clinical research as the prospective selection of a study population in which detection of a treatment effect (if one is in fact present) is more likely than it would be in an unselected population [32]. The aim here is to increase the efficiency of a treatment and support a more precision medicine in heterogeneous populations.

The selection of patients could be based on a known pathway biomarker or be unsupervised (i.e., empiric) in case of unknown mechanisms (e.g., a subphenotyping/endotyping approach) [33]. This approach is distinct from prognostic enrichment whereby higher-risk individuals are enrolled with the expectation of increasing the event rate and, consecutively, statistical power [34].

However, as demonstrated in the IMPRESS trial of AMICS that specifically enrolled high-risk cardiogenic shock individuals (> 90% of whom were comatose after cardiac arrest), high-risk individuals may not be more likely to respond to a tested intervention if they have competing mortality risks that are not modified by the intervention [35].

Re-analyzing results of major ‘neutral’ treatment trials in circulatory shock from the perspective of biological mechanisms may allow identification of salutary effects of treatments in subclasses of patients (i.e., treatable traits) [36–38]. It should be noted that this framework considers possibility effect-based (or predictive) heterogeneity of treatment effect. An alternative approach is risk-based (or prognostic) heterogeneity of treatment effect. Both frameworks may have value in critically ill populations [34], although this review focused on effect-based (predictive) markers given their closer tie to underlying biologic host response. Indeed, these approaches are not mutually exclusive if a relevant biological pathway targeted by a specific therapy is itself prognostic.

## Predictive enrichment in circulatory shock by using biomarker-guided therapy

Predictive enrichment can be achieved by classifying patients using a biomarker linked to the tested intervention. In the EUPHRATES trial, Dellinger et al*.* assessed the use of high-affinity polymyxin B hemoperfusion in patients with septic shock to remove bacterial endotoxin from the circulation through selective adsorption [39].

The authors randomized only patients with an endotoxin activity assay level of 0.60 or higher. Polymyxin B hemoperfusion compared with sham hemoperfusion did not significantly decrease 28-day mortality among the randomized patients. In a post hoc analysis of the EUPHRATES trial including high severity of illness (Multiple Organ Dysfunction Score (MODS) > 9) and an endotoxin activity level between 0.6 and 0.89, Polymyxin B hemoperfusion use was associated with an absolute mortality reduction compared to sham patients of 10.7% at 28 days [40]. These results are supported by benefit across secondary end points such as change in MAP from baseline to day 3 and days alive and free of mechanical ventilation.

In a post hoc analysis of the ‘neutral’ Sepsis Coagulopathy Asahi Recombinant LE Thrombomodulin trial (SCARLET), the authors reported lower mortality in patients with septic shock associated coagulopathy and elevated coagulation markers (prothrombin fragment 1.2, thrombin–antithrombin complex, d-dimer) treated with thrombomodulin, suggesting this coagulation pattern could be used to select patients most likely to respond [41].

In another retrospective analysis of the phase III randomized interleukin-1 receptor antagonist trial in Sepsis patients with multiorgan dysfunction syndrome and/or shock, the authors reported in the subclass of patients with macrophage activation syndrome (i.e., high circulating markers of hepatobiliary dysfunction and disseminated intravascular coagulation) an association of IL-1 receptor blockade with significant improvement in survival at 28 days [42]. This signal of improved clinical outcomes was not detected in the original study at the level of the whole population (septic patients with and without macrophage activation syndrome) [43].

A post hoc analysis of patients enrolled in the ATHOS-3 trial (Angiotensin (AT) II for the Treatment of High-Output Shock) [44], tested the hypothesis that there is a disturbance in the renin–angiotensin–aldosterone system (i.e., insufficiency in the angiotensin-converting enzyme activity) in catecholamine-resistant vasodilatory shock from different etiologies (predominantly sepsis).

The authors reported that in vasodilatory shock patients with renin concentrations higher than the study population median, angiotensin II infusion significantly reduced 28-day mortality when compared with placebo [45] which was not the case in the original ATHOS-3 study at the level of the entire study population [44]. Importantly, patients with high renin levels were also more likely to respond favorably to AT-II infusion in terms of blood pressure response with a greater likelihood of renal recovery in patients with AKI requiring renal replacement therapy and high renin levels who received AT-II [46].

The authors hypothesized that the inflammatory host response in circulatory shock may reduce angiotensin-converting enzyme activity, which may lead to decreased conversion of AT-I to AT-II and conversion of AT-I to vasodilatory AT degradation peptides causing persistent hypotension and high renin-levels [45]. They concluded that serum renin concentration could be used to identify patients with catecholamine-resistant vasodilatory shock who may benefit from treatment with synthetic angiotensin II.

In the AdrenOSS-2 phase 2a biomarker-guided trial, Laterre et al. investigated a non-neutralizing adrenomedullin antibody (adrecizumab) in septic shock patients with high adrenomedullin [47]. The primary endpoint of the trial was achieved as adrecizumab was well tolerated and among the secondary endpoints the reduction in Sequential Organ Failure Assessment (SOFA) Score was significantly higher (i.e., resolution of organ dysfunction) in the treatment group compared to placebo. In a more recent work, the same research group reported that in septic shock patients with high adrenomedullin levels included in AdrenOSS-2, a further *post hoc* enrichment strategy based on circulating dipeptidyl peptidase 3 (cDPP3) (a metallopeptidase involved in the metabolism of cardiovascular and inflammatory mediators that exert a direct negative inotropic action) may indicate that therapeutic efficacy is most important (28-day mortality) in patients with lower cDPP3 levels [48]. The same approach as the original AdrenOSS-2 study was applied in the ACCOST-HH trial including cardiogenic shock patients. Adrecizumab was well tolerated but did not decrease the need for mechanical circulatory support (primary endpoint) or improve survival at days 30 and 90 [49]**.**

## Predictive enrichment in circulatory shock by using subphenotyping

The pathways underlying different circulatory shock syndromes are complex and treatment responses are multifactorial, necessitating multiple biomarkers to identify a significant amount of the variability in response (i.e., modeling biological heterogeneity, interplay between different pathways). Limiting ourselves to known biomarkers may not advance much-needed discovery which can instead be promoted using unsupervised clustering approaches (i.e., agnostic to outcome).

Patients can be classified using multiple readily available clinical and biological data or more sophisticated high-dimensional biomarkers (i.e., omics-based biomarkers). The identification of subclasses/subphenotypes using clustering and unsupervised machine learning algorithms (e.g., hierarchical clustering, *k*-means clustering, latent class analysis) may allow the identification of distinct mechanistic signatures underlying the heterogeneous circulatory shock syndromes and the discovery of candidate biotargets (i.e., actionable biomarkers) [50–52].

In Seymour et al. study, simulation models suggested that the four phenotypes (α, β, γ, and δ) identified using unsupervised clustering demonstrated proof-of-concept in support for molecular endotypes underscoring treatment effects in septic shock patients [15]. The estimated treatment effects were variable across the different phenotypes with a significant interaction between the tested treatment and phenotypes in the ProCESS trial (early goal-directed therapy in septic shock patients). In the same trial, the chance of finding benefit with early goal-directed therapy increased when the α phenotype (patients with less organ dysfunction) represented most of the studied population. Conversely, when the δ phenotype (elevated serum lactate levels, elevated levels of transaminases, and hypotension) was increased, there was a higher chance of finding that early goal-directed therapy was harmful.

In a post hoc analysis of the VANISH trial including patients with septic shock [53], genome-wide gene expression profiling was performed and the SRS1 (immunosuppressed) and SRS2 (immunocompetent) endotypes were replicated by a previously established model using seven discriminant genes in the study of Davenport et al. [17]. The authors reported an interaction between SRS endotype and assignment to hydrocortisone or placebo. Hydrocortisone use was associated with increased mortality in septic shock patients assigned in the SRS2 endotype.

In a retrospective analysis of the PROPPR randomized trial including severe trauma patients with hemorrhagic shock which compared transfusion of plasma, platelets, and red blood cells in a 1:1:1 ratio to a 1:1:2 ratio regarding mortality, the investigators applied latent class analysis to identify two trauma subphenotypes (TS-1 and TS-2). They used 36 circulating markers of inflammation, endothelial dysfunction, and coagulation measured prior to patients’ randomization. In patients assigned to TS-2 (lower plasma concentrations of IL-8 and TNF-α), a 1:1:1 transfusion ratio was associated with significantly reduced risk for 30-day mortality compared to a 1:1:2 approach when adjusted for age, sex, injury severity, and injury mechanism. There was no difference in mortality by treatment assignment when the patients were stratified by severity of illness severity or injury mechanism [54].

A summary of the aforesaid circulatory shock studies with predictive enrichment is represented in Table 1.

**Table 1:** Characteristics of the main circulatory shock studies with predictive enrichment

Study (year)	Design	Study population	Population size	Predictive enrichment strategy	Variables used for enrichment	Intervention	Main results
Klein et al. (2018) [40]	Post hoc analysis of the EUPHRATES trial	Septic shock patients with MODS	*N* = 194 (original study N = 450)	Biomarker-guided therapy	Endotoxin activity level between 0.6 and 0.89	Polymyxin B hemoperfusion to remove bacterial endotoxin	Associated with an absolute mortality reduction at 28 days Secondary outcomes: benefit change in MAP from baseline to day 3 and days alive and free of mechanical ventilation
Levi et al. (2020) [41]	Post hoc analysis of the SCARLET trial	Septic shock associated coagulopathy	*N* = 800	Biomarker-guided therapy	Elevated coagulation markers (prothrombin fragment 1.2, thrombin–antithrombin complex, d-dimer)	Recombinant human soluble thrombomodulin	Associated with all-cause mortality reduction at 28 days
Shakoory et al. (2016) [42]	Post hoc analysis of the phase III randomized interleukin-1 receptor antagonist trial	Sepsis patients with MODS and/or shock	*N* = 43 (original study N = 763)	Biomarker-guided therapy	Hepatobiliary dysfunction and disseminated intravascular coagulation as features of macrophage activation syndrome	Anakinra (recombinant interleukin-1 receptor antagonist)	Significant improvement in survival at 28 days
Bellomo et al. (2020) [45]	Post hoc analysis of the ATHOS-3 trial	catecholamine-resistant vasodilatory shock patients (sepsis, pancreatitis, post operative vasoplegia)	*N* = 321	Biomarker-guided therapy	Serum renin concentrations	Angiotensin II	In patients with renin concentrations above the study population median, angiotensin II significantly reduced 28-day mortality
Laterre et al. (2020) [47]	The AdrenOSS-2 phase 2a biomarker-guided trial	Septic shock with high Bio-ADM patients	*N* = 301	Biomarker-guided therapy	Circulating Bio-ADM (> 70 pg/mL)	Adrecizumab (a humanized monoclonal adrenomedullin antibody)	Primary endpoint: good tolerance of adrecizumab Secondary endpoint: the reduction in *SOFA score* was significantly higher in the treatment group vs placebo
Van Lier et al. (2022) [49]	*Post hoc* analysis of the AdrenOSS-2 trial	Septic shock with high Bio-ADM patients	*N* = 249	Biomarker-guided therapy	*Post hoc* enrichment strategy based on low cDPP3 (< 50 ng/mL)	Adrecizumab (a humanized monoclonal adrenomedullin antibody)	In patients with low cDPP3 levels, Adrecizumab significantly reduced 28-day mortality
Karakas et al. (2022) [50]	The ACCOST-HH biomarker-guided trial	Cardiogenic shock with high Bio-ADM patients	*N* = 150	Biomarker-guided therapy	Circulating Bio-ADM (> 70 pg/mL)	Adrecizumab (a humanized monoclonal adrenomedullin antibody)	Adrecizumab was well tolerated but did not decrease the need for cardiovascular organ support (primary endpoint) or improve survival at days 30 and 90
Seymour et al. (2019) [15]	*Post hoc* analysis of observational studies and clinical trials	Sepsis and septic shock patients	*N* = 20 189 (the SENECA derivation cohort)	Phenotyping (latent class analysis, consensus K-means clustering)	29 readily available clinical and biological variables on admission	Early goal-directed therapy in septic shock patients (ProCESS trial)	The estimated treatment effects were variable across the different identified four phenotypes (α, β, γ, and δ) with a significant interaction between early goal-directed therapy and phenotypes in the ProCESS trial
Antcliffe et al. (2019) [53]	*Post hoc* analysis of the VANISH trial	Septic shock patients	*N* = 176	Phenotyping (hierarchical clustering)	Genome-wide gene expression profiling (transcriptomic data)	Hydrocortisone	Two endotypes were identified: SRS1 (immunosuppressed) and SRS2 (immunocompetent). Hydrocortisone use was associated with increased mortality in septic shock patients assigned in the SRS2 endotype
Thau et al. (2022) [54]	*Post hoc* analysis of the PROPPR trial	Severe trauma patients with hemorrhagic shock	*N* = 478	Phenotyping (latent class analysis)	36 plasma biomarkers of inflammation, endothelial dysfunction, and coagulation	Transfusion of plasma, platelets, and red blood cells in a 1:1:1 vs a 1:1:2 Ratio	Two trauma subphenotypes (TS-1 and TS-2) were identified. In patients assigned to TS-2 (lower plasma concentrations of IL-8 and TNF-α), a 1:1:1 transfusion ratio was associated with significantly reduced risk for 30-day mortality

MAP, mean arterial pressure; Bio-ADM, biologically active adrenomedullin; SOFA score, Sequential Organ Failure Assessment (*SOFA*) *score*; cDPP3, circulatory dipeptidyl peptidase 3; IL-8, interleukin 8; TNF-α, tumor necrosis factor α

## Future directions and challenges

The identification of distinct host-response subclasses/subphenotypes may further inform mechanisms of persistent organ dysfunction and enable prognostic and predictive enrichment in circulatory shock regardless of the etiology or clinical classification. Secondary analyses of major critical care clinical trials and measurement of biomarkers in longitudinal biobanked samples to identify signals for benefit in a biologically defined subclass could evaluate whether the biological profiles are just capturing different points along patients’ trajectory towards a final common pathway, whether the biological signature is the same for all interventions studied or specific to a targeted intervention and whether the same heterogeneity of treatment effect is seen in other etiologies of circulatory shock.

These analyses may support future randomized controlled trials of personalized therapies in circulatory shock patients in which patients are prospectively enrolled based on a biological signature rather than on a non-specific clinical syndrome, such as sepsis, cardiogenic shock, or major trauma that invites empiric therapy. This approach (i.e., biomarker-guided therapy or subphenotype-guided therapy) may be a key step to improve translation of research findings to the bedside for a more personalized critical care medicine. As a result, for example, we could be talking about “renin and/or bio-adrenomedullin-driven shock”, etc., rather than clinically defined “syndrome/etiology” models. We believe that regardless of the triggering etiology, the development of refractory shock and organ failure likely occurs via multiple overlapping conserved pathophysiological mechanisms, and their persistence over time which might be targets for therapy.

Identifying distinct biological patterns is promising, but it does not guarantee the distinction of specific pathophysiological processes with causal links to intervention effects that might ultimately allow a personalization of treatment in circulatory shock patients. Diverse influences on these biomarkers are present, and it is unproven how well these candidate markers integrate diverse clinical, genetic, pathophysiologic, and treatment influences. Nevertheless, there is a theoretical advantage that they may summarize these diverse, ‘upstream’ influences on some extent and represent biologic indicators closer to the patient phenotype.

To minimize the risk of categorizing the studied population differently (i.e., move from clinical to biological classes) without capturing the complexity of underlying biological mechanisms, we think it is important to consider the following points. The use of a multi-biomarker approach reflecting diverse pathways involved in circulatory shock in humans (e.g., inflammation, immunosuppression, endothelial dysfunction, organ injury among others) may support comprehensive profiling. Ideally, this approach can be coupled with modeling the dynamic interplay between different actionable biomarkers using unsupervised machine learning (e.g., latent class analysis) instead of a single-biomarker approach with a single cutoff.

Without considering personalized host responses, dozens of promising targeted molecular therapies and efforts to individualize risk prediction have failed to reduce mortality. This parallels oncology clinical research with a high response rate of successful targeted therapies in biomarker-driven patient selection evaluated in early clinical trials [55–57].

**Fig. 1:**
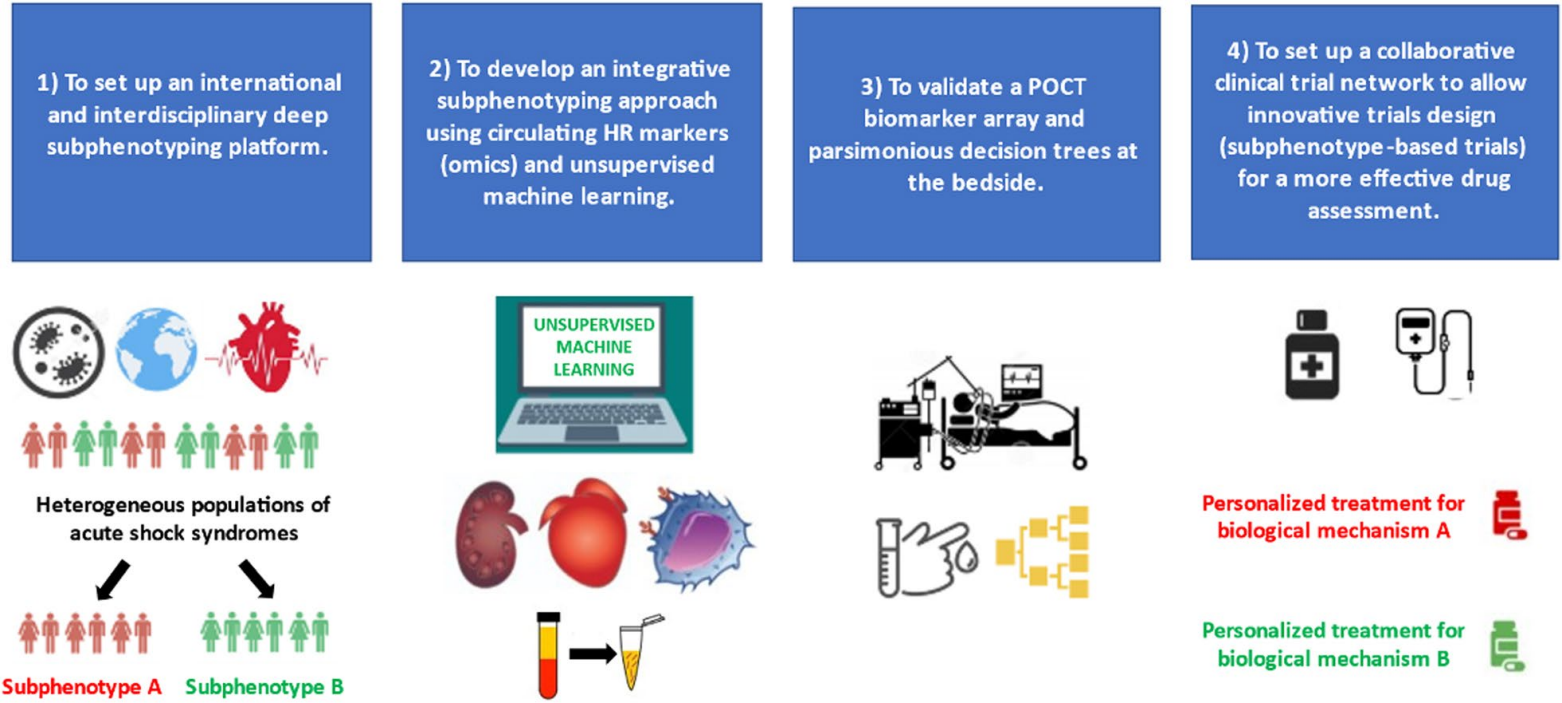
A suggested roadmap for a mechanistic subphenotyping approach in the circulatory shock syndrome (e.g., severe trauma, cardiogenic shock, sepsis). HR, host response; POCT, point-of-care testing

We suggest the following roadmap as a strategy to advance research in circulatory shock (Fig. 1):

First to set up a collaborative, multicenter and interdisciplinary deep subphenotyping platform centered on the biological/molecular drivers of circulatory shock to identify and largely validate distinct mechanistic signatures in circulatory shock (i.e., endotypes). The French and European Outcome Registry in Intensive Care Units (FROG-ICU) is an example of a cohort with a biobank and molecular trait mapping of circulatory shock patients with different etiologies, under one “roof” supporting feasibility (trials.gov identifier: NCT01367093) [58, 59].

**Fig. 2:**
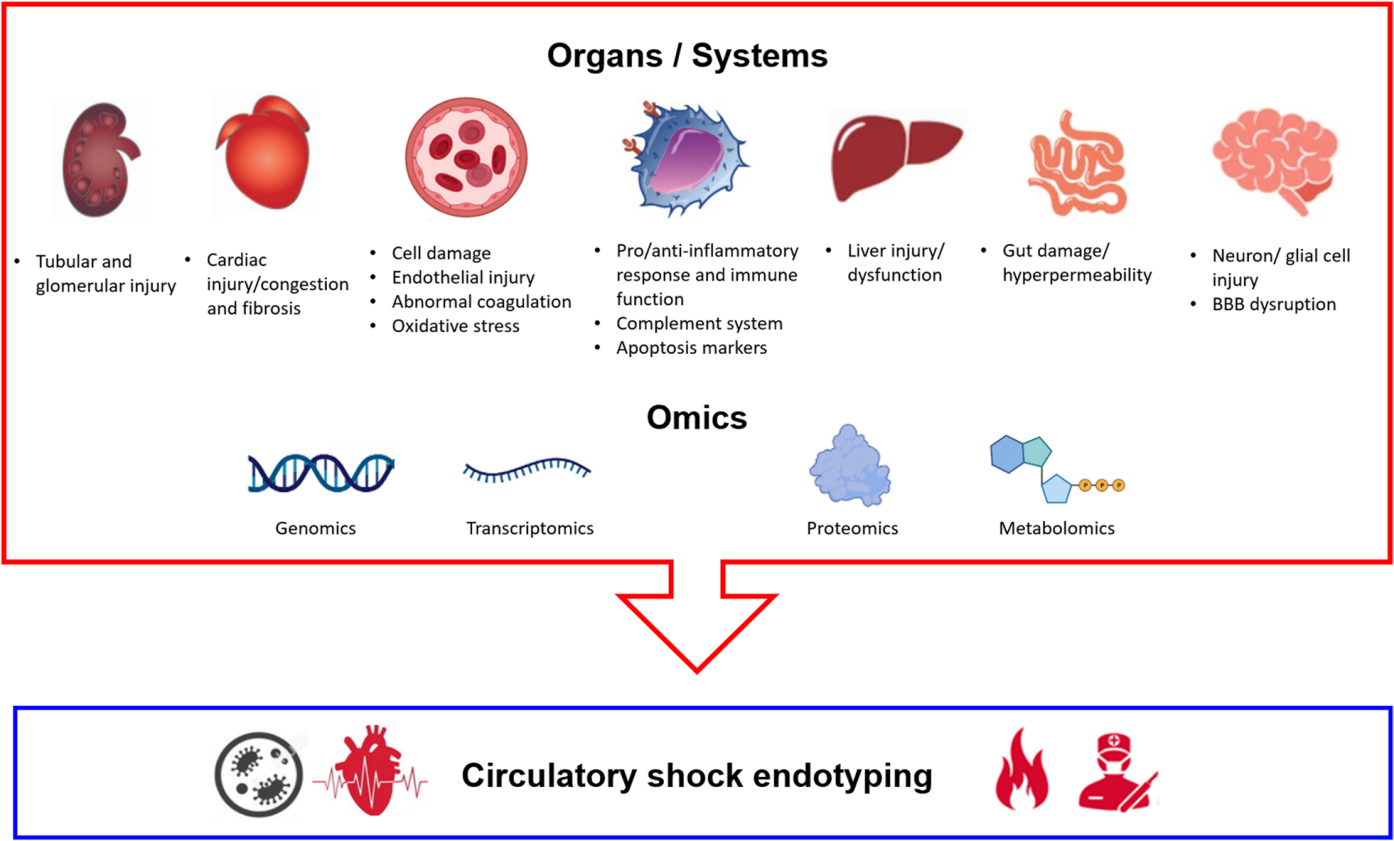
A suggested panel of groups of biomarkers for circulatory shock syndrome endotyping

Second, coupling discovery research with clinical trials and develop an integrative subphenotyping approach using unsupervised machine learning and biomarker data to inform effective new therapies in future clinical trials. This may allow signal enrichment and noise reduction and help decrease neutral circulatory shock clinical trials. Developing a harmonized strategy is fundamental with minimal criteria of groups of candidate biomarkers such as inflammatory, immune dysfunction, endothelial injury, organ dysfunction (e.g., renal, cardiac, neurological, gut), abnormal coagulation, cell damage and oxidative stress for circulatory shock endotyping. A suggestion of a panel of groups of biomarkers for circulatory shock endotyping is represented in Fig. 2.

Implementation of biomarker-stratified adaptive clinical trial designs is likely to be an important mechanism to facilitate this approach. Adaptive clinical trials may offer opportunities to better accommodate the possibility of heterogeneous treatment effects into the trial design prospectively—a tool which may better support individualization of clinical care based on host-response profile [60, 61]. Third, to validate a point-of-care biomarker array and parsimonious decision trees at the bedside to classify patients in distinct subphenotypes [62].

Many obstacles need to be overcome to implement biological subclasses/subphenotypes into clinical trials design and daily practice at the bedside. First the important heterogeneity of studies using different clustering approaches and biomarkers. Second, the stability/evolution and overlap of the identified subphenotypes and the interaction between comorbid illnesses and acute subphenotypes which makes it difficult to understand the overlap between subclasses with a poor replicability. Third, the timely serial assignment of subphenotype at the bedside using a simplified clustering/classification algorithm and rapid real-time assays in the ICU [63]. Last and not least, the response to treatment is not only driven by “patient heterogeneity”, but also the severity of stage of the disease/insult—for example in the neutral ALBIOS trial assessing albumin administration in septic patients, only a subgroup of patients with circulatory shock showed a benefit from this resuscitation strategy [64].

## Conclusion

Emerging translational evidence highlights existing heterogeneity regarding the underlying host response between and within circulatory shock syndromes on one hand and demonstrates the existence of potential common ‘treatable traits’ within subgroups of patients across different forms of shock on the other hand. Further research should focus on these host-response pathways to shift from a cause–consequence model towards a host-response subphenotype model. This could pave the way towards personalized critical care medicine.

### Take-home messages

- Traditional shock subgroups defined clinically on basis of etiology and hemodynamic features are incorrectly considered homogeneous entities. Emerging translational evidence highlights the existing heterogeneity regarding the underlying host response between and within circulatory shock syndromes.
- Accepting the results of neutral clinical trials without taking into consideration the heterogeneity of underlying biological mechanisms and the existence of distinct host-response patterns is a missed opportunity to discover subclasses of patients that may benefit from targeted treatments.
- Identifying host-response patterns may provide new insights regarding the pathophysiology of circulatory shock and pave the way towards the development of innovative biomarkers and targets of pharmacological therapies. This may allow predictive enrichment to define personalized treatments (biomarker-driven or endotype-driven patient selection in future clinical trials) and ultimately transform current research and care.

## Data Availability

Not applicable.

## References

[1] Santacruz CA, Pereira AJ, Celis E, Vincent J-L (2019). Which multicenter randomized controlled trials in critical care medicine have shown reduced mortality? A systematic review. Crit Care Med.

[2] Arrigo M, Price S, Baran DA (2021). Optimising clinical trials in acute myocardial infarction complicated by cardiogenic shock: a statement from the 2020 critical care clinical trialists workshop. Lancet Respir Med.

[3] Tyler JM, Brown C, Jentzer JC (2022). Variability in reporting of key outcome predictors in acute myocardial infarction cardiogenic shock trials. Catheter Cardiovasc Interv.

[4] Marshall JC (2014). Why have clinical trials in sepsis failed?. Trends Mol Med.

[5] Vincent J-L, De Backer D (2013). Circulatory shock. N Engl J Med.

[6] Cecconi M, De Backer D, Antonelli M (2014). Consensus on circulatory shock and hemodynamic monitoring. Task force of the European Society of Intensive Care Medicine. Intensive Care Med.

[7] Vincent J-L (2022). Current sepsis therapeutics. EBioMedicine.

[8] Johansson PI, Stensballe J, Ostrowski SR (2017). Shock induced endotheliopathy (SHINE) in acute critical illness-a unifying pathophysiologic mechanism. Crit Care.

[9] Luperto M, Zafrani L (2022). T cell dysregulation in inflammatory diseases in ICU. Intensive Care Med Exp.

[10] Binnie A, Tsang JLY, Hu P (2020). Epigenetics of sepsis. Crit Care Med.

[11] Maslove DM, Tang B, Shankar-Hari M (2022). Redefining critical illness. Nat Med.

[12] Lawler PR, Mehra MR (2018). Advancing from a “hemodynamic model” to a “mechanistic disease-modifying model” of cardiogenic shock. J Heart Lung Transpl.

[13] Cusack R, Leone M, Rodriguez AH, Martin-Loeches I (2022). Endothelial damage and the microcirculation in critical illness. Biomedicines.

[14] Braga D, Barcella M, Herpain A (2019). A longitudinal study highlights shared aspects of the transcriptomic response to cardiogenic and septic shock. Crit Care.

[15] Seymour CW, Kennedy JN, Wang S (2019). Derivation, validation, and potential treatment implications of novel clinical phenotypes for sepsis. JAMA.

[16] Cummings MJ, Bakamutumaho B, Price A (2022). Multidimensional analysis of the host response reveals prognostic and pathogen-driven immune subtypes among adults with sepsis in Uganda. Crit Care.

[17] Davenport EE, Burnham KL, Radhakrishnan J (2016). Genomic landscape of the individual host response and outcomes in sepsis: a prospective cohort study. Lancet Respir Med.

[18] Zweck E, Thayer KL, Helgestad OKL (2021). Phenotyping cardiogenic shock. J Am Heart Assoc.

[19] Jentzer JC, Soussi S, Lawler PR (2022). Validation of cardiogenic shock phenotypes in a mixed cardiac intensive care unit population. Catheter Cardiovasc Interv.

[20] Toma A, Dos Santos C, Burzyńska B (2022). Diversity in the expressed genomic host response to myocardial infarction. Circ Res.

[21] Scolari FL, Abelson S, Brahmbhatt DH (2022). Clonal haematopoiesis is associated with higher mortality in patients with cardiogenic shock. Eur J Heart Fail.

[22] Brakenridge SC, Wang Z, Cox M (2021). Distinct immunologic endotypes are associated with clinical trajectory after severe blunt trauma and hemorrhagic shock. J Trauma Acute Care Surg.

[23] Cyr A, Zhong Y, Reis SE (2021). Analysis of the plasma metabolome after trauma, novel circulating sphingolipid signatures, and in-hospital outcomes. J Am Coll Surg.

[24] Mebazaa A, Geven C, Hollinger A (2018). Circulating adrenomedullin estimates survival and reversibility of organ failure in sepsis: the prospective observational multinational Adrenomedullin and Outcome in Sepsis and Septic Shock-1 (AdrenOSS-1) study. Crit Care.

[25] Tolppanen H, Rivas-Lasarte M, Lassus J (2017). Adrenomedullin: a marker of impaired hemodynamics, organ dysfunction, and poor prognosis in cardiogenic shock. Ann Intensive Care.

[26] Fisher J, Douglas JJ, Linder A (2016). Elevated plasma angiopoietin-2 levels are associated with fluid overload, organ dysfunction, and mortality in human septic shock. Crit Care Med.

[27] Uhlich RM, Richter RP, Hu PJ (2020). Temporal dysregulation of the angiopoietin-2/-1 ratio after trauma and associations with injury characteristics and outcomes. Shock.

[28] Pöss J, Fuernau G, Denks D (2015). Angiopoietin-2 in acute myocardial infarction complicated by cardiogenic shock–a biomarker substudy of the IABP-SHOCK II-Trial. Eur J Heart Fail.

[29] Xiao W, Mindrinos MN, Seok J (2011). A genomic storm in critically injured humans. J Exp Med.

[30] Venet F, Textoris J, Blein S (2022). Immune profiling demonstrates a common immune signature of delayed acquired immunodeficiency in patients with various etiologies of severe injury. Crit Care Med.

[31] Chen T, Conroy J, Wang X (2022). The independent prognostic value of global epigenetic alterations: an analysis of single-cell ATAC-seq of circulating leukocytes from trauma patients followed by validation in whole blood leukocyte transcriptomes across three etiologies of critical illness. EBioMedicine.

[32] Research C for DE (2019) Enrichment strategies for clinical trials to support approval of human drugs and biological products. In: U.S. Food and Drug Administration. https://www.fda.gov/regulatory-information/search-fda-guidance-documents/enrichment-strategies-clinical-trials-support-approval-human-drugs-and-biological-products. Accessed 4 Mar 2023

[33] Lawler PR, Fan E (2018). Heterogeneity and phenotypic stratification in acute respiratory distress syndrome. Lancet Respir Med.

[34] Iwashyna TJ, Burke JF, Sussman JB (2015). Implications of heterogeneity of treatment effect for reporting and analysis of randomized trials in critical care. Am J Respir Crit Care Med.

[35] Ouweneel DM, Eriksen E, Sjauw KD (2017). Percutaneous mechanical circulatory support versus intra-aortic balloon pump in cardiogenic shock after acute myocardial infarction. J Am Coll Cardiol.

[36] De Backer D, Biston P, Devriendt J (2010). Comparison of dopamine and norepinephrine in the treatment of shock. N Engl J Med.

[37] Ranieri VM, Thompson BT, Barie PS (2012). Drotrecogin alfa (activated) in adults with septic shock. N Engl J Med.

[38] Mathew R, Di Santo P, Jung RG (2021). Milrinone as compared with dobutamine in the treatment of cardiogenic shock. N Engl J Med.

[39] Dellinger RP, Bagshaw SM, Antonelli M (2018). Effect of targeted polymyxin B hemoperfusion on 28-day mortality in patients with septic shock and elevated endotoxin level: the EUPHRATES randomized clinical trial. JAMA.

[40] Klein DJ, Foster D, Walker PM (2018). Polymyxin B hemoperfusion in endotoxemic septic shock patients without extreme endotoxemia: a post hoc analysis of the EUPHRATES trial. Intensive Care Med.

[41] Levi M, Vincent J-L, Tanaka K (2020). Effect of a recombinant human soluble thrombomodulin on baseline coagulation biomarker levels and mortality outcome in patients with sepsis-associated coagulopathy. Crit Care Med.

[42] Shakoory B, Carcillo JA, Chatham WW (2016). Interleukin-1 receptor blockade is associated with reduced mortality in sepsis patients with features of macrophage activation syndrome: reanalysis of a prior phase III trial. Crit Care Med.

[43] Opal SM, Fisher CJ, Dhainaut JF (1997). Confirmatory interleukin-1 receptor antagonist trial in severe sepsis: a phase III, randomized, double-blind, placebo-controlled, multicenter trial. The Interleukin-1 receptor antagonist sepsis investigator group. Crit Care Med.

[44] Khanna A, English SW, Wang XS (2017). Angiotensin II for the treatment of vasodilatory shock. N Engl J Med.

[45] Bellomo R, Forni LG, Busse LW (2020). Renin and survival in patients given angiotensin II for catecholamine-resistant vasodilatory shock. a clinical trial. Am J Respir Crit Care Med.

[46] Tumlin JA, Murugan R, Deane AM (2018). Outcomes in patients with vasodilatory shock and renal replacement therapy treated with intravenous angiotensin II. Crit Care Med.

[47] Laterre P-F, Pickkers P, Marx G (2021). Safety and tolerability of non-neutralizing adrenomedullin antibody adrecizumab (HAM8101) in septic shock patients: the AdrenOSS-2 phase 2a biomarker-guided trial. Intensive Care Med.

[48] van Lier D, Picod A, Marx G (2022). Effects of enrichment strategies on outcome of adrecizumab treatment in septic shock: post-hoc analyses of the phase II adrenomedullin and outcome in septic shock 2 trial. Front Med (Lausanne).

[49] Karakas M, Akin I, Burdelski C (2022). Single-dose of adrecizumab versus placebo in acute cardiogenic shock (ACCOST-HH): an investigator-initiated, randomised, double-blinded, placebo-controlled, multicentre trial. Lancet Respir Med.

[50] Soussi S, Collins GS, Jüni P (2021). Evaluation of biomarkers in critical care and perioperative medicine: a clinician’s overview of traditional statistical methods and machine learning algorithms. Anesthesiology.

[51] Komorowski M, Green A, Tatham KC (2022). Sepsis biomarkers and diagnostic tools with a focus on machine learning. EBioMedicine.

[52] Jentzer JC, Rayfield C, Soussi S (2022). Machine learning approaches for phenotyping in cardiogenic shock and critical illness. JACC Adv.

[53] Antcliffe DB, Burnham KL, Al-Beidh F (2019). Transcriptomic signatures in sepsis and a differential response to steroids. From the VANISH randomized trial. Am J Respir Crit Care Med.

[54] Thau MR, Liu T, Sathe NA, et al (2022) Latent class analysis in a trauma cohort with hemorrhagic shock identifies two distinct sub-phenotypes with a differential treatment response to blood transfusion ratios. In: Abstracts of the American Thoracic Society International Conference, San Francisco, 13–18 May 2022

[55] Dienstmann R, Rodon J, Tabernero J (2013). Biomarker-driven patient selection for early clinical trials. Curr Opin Oncol.

[56] Flaherty KT, Puzanov I, Kim KB (2010). Inhibition of mutated, activated BRAF in metastatic melanoma. N Engl J Med.

[57] Kopetz S, Grothey A, Yaeger R (2019). Encorafenib, Binimetinib, and Cetuximab in BRAF V600E-mutated colorectal cancer. N Engl J Med.

[58] Deniau B, Picod A, Van Lier D (2022). High plasma dipeptidyl peptidase 3 levels are associated with mortality and organ failure in shock: results from the international, prospective and observational FROG-ICU cohort. Br J Anaesth.

[59] Soussi S, Sharma D, Jüni P (2022). Identifying clinical subtypes in sepsis-survivors with different one-year outcomes: a secondary latent class analysis of the FROG-ICU cohort. Crit Care.

[60] Lawler PR (2023). Models for evidence generation during the COVID-19 pandemic: new opportunities for clinical trials in cardiovascular medicine. Circulation.

[61] Lawler PR, Hochman JS, Zarychanski R (2022). What are adaptive platform clinical trials and what role may they have in cardiovascular medicine?. Circulation.

[62] Wong HR, Sweeney TE, Lindsell CJ (2017). Simplification of a septic shock endotyping strategy for clinical application. Am J Respir Crit Care Med.

[63] Reddy K, Sinha P, O’Kane CM (2020). Subphenotypes in critical care: translation into clinical practice. Lancet Respir Med.

[64] Caironi P, Tognoni G, Masson S (2014). Albumin replacement in patients with severe sepsis or septic shock. N Engl J Med.

